# Mental health and COVID-19 vaccine hesitancy among health-related university students: a cross-sectional multi-center study

**DOI:** 10.1186/s41182-025-00751-3

**Published:** 2025-05-19

**Authors:** Thanawat Khongyot, Amy Takyi, Dympna Siysila Ndzeshang, Karl Gwei Njuwa Fai, Tin Zar Win, Latdavanh Vorlasane, Gibson Omwansa Javes, Satoshi Kaneko, Taeko Moriyasu

**Affiliations:** 1 School of Pharmacy, Walailak University, Nakhon Si Thammarat, Thailand; 2 Health Information Officer, Apam, Central Region Ghana; 3 School of Tropical Medicine and Global Health, Nagasaki University, Nagasaki, Japan; 4 Epicentre, Médecins Sans Frontières, Yaoundé, Cameroon; 5 Savannakhet Provincial Hospital, Savannakhet, Lao PDR; 6 Institute of Tropical Medicine, Nagasaki University, Nagasaki, Japan; 7 Office for Global Relations, Nagasaki University, 1-14 Bunkyo, Nagasaki, 852-8521 Japan

**Keywords:** COVID-19 vaccine, Coronavirus disease, Mental health, University student, Vaccine hesitancy

## Abstract

**Background:**

The mental health problems during the coronavirus disease 2019 (COVID-19) pandemic may have influenced their decision to receive the COVID-19 vaccine among health-related university students, with potential differences across countries. This study elucidated the association between mental health and COVID-19 vaccine hesitancy of health-related university students in Thailand, Laos, and Japan. We additionally examined the other factors that might relate to COVID-19 vaccine hesitancy.

**Methods:**

The study conducted an online survey from February 4 to 27, 2021, among undergraduate students enrolled in health-related programs at University of Health Sciences (Lao PDR), Walailak University (Thailand), and Nagasaki University (Japan) using a non-probability convenience sampling method. The data were analyzed using multiple logistic regression to identify associations between mental health and self-reported COVID-19 vaccine hesitancy and other potential factors.

**Results:**

This study analyzed data from 841 students. Japanese students attended hybrid classes (82.45%), while those in Laos and Thailand had entirely online courses. All mental health assessment scores (depression, anxiety, and stress) were higher in Thailand and Laos compared to Japan. Students with very high-stress scores had the highest odds of vaccine hesitancy (aOR 2.67, 95% CI 1.45–4.93). Fear of COVID-19 increased hesitancy, while unbelief in vaccine protection significantly increased it (aOR 2.59, 95% CI 1.86–3.59). Females displayed about two times greater hesitancy (adjusted odds ratio, aOR 2.43, 95% CI 1.68–3.51), which correlated with higher mental health scores.

**Conclusions:**

We highlighted a significant association between mental health and self-report COVID-19 vaccine hesitancy. Interventions, including tailored support, awareness campaigns, and psychological services, can foster trust and vaccine uptake.

**Supplementary Information:**

The online version contains supplementary material available at 10.1186/s41182-025-00751-3.

## Background

Current control measures for Coronavirus Disease 2019 (COVID-19) seek to decrease and eventually stop transmission while maintaining an optimal balance between the health system, economy, and society. COVID-19 vaccination remains a basis in the global response to the COVID-19 pandemic. As of 2023, according to World Health Organization (WHO) data, COVID-19 vaccination coverage varied across countries. In Thailand, 46% of the population had received at least one booster dose, compared to 34% in Lao PDR and 69% in Japan. Primary series vaccination coverage (based on each vaccine product dosing schedule) was 78% in both Thailand and Lao PDR, and 82% in Japan. This information reflects differing levels of vaccine uptake coverage [1].

Moreover, mental health issues should not be overlooked during the COVID-19 pandemic. Some study has demonstrated an increase in the incidence of mental health issues among medical students who study in college and university during the COVID-19 pandemic [2]. The younger age group has a higher risk of mental health problems. Each country may affect people differently, especially mental-related problems, such as depression, anxiety, and stress [2, 3]. In Thailand, a study found depression in 21.4% and anxiety in 7.8% of university students [4], while another reported a 28.8% depression rate among Thai medical students, with a link to internet addiction [5, 6]. A 2014 nationwide study in Japan showed poor mental health in 36.6% of male and 48.8% of female medical students [7]. Another Japanese study found 17.3% of high school students had depression and 19.0% had anxiety, significantly associated with long commuting times and high electronic device use [8]. While mental health data among Lao undergraduate students remains limited.

Mental health problems might affect the decision to get the vaccine [9]. The level of mental health problems may delay the use of the COVID-19 vaccine in different countries. Therefore, this study explored the COVID-19 vaccine hesitancy and mental health issues in Thailand, Laos, and Japan to understand the situation and recommend timely, practical solutions to improve vaccine hesitancy accurately.

## Methods

### Ethical approval and registration

Ethical approvals were obtained from the following committees prior to data collection: Japan: The Ethical Committee of Institute of Tropical Medicine, Nagasaki University (Approval No. 210225256); Thailand: The Ethical Committee of Walailak University (Approval No. WUEC-21-138-01); Lao PDR: Ministry of Health University of Health Sciences Research Ethics Committee (Approval No. 203/REC).

### Study setting

This study was conducted to compare the characteristics of mental issues among undergraduate students at three universities: Nagasaki University, Japan; Walailak University, Thailand; and University of Health Science, Lao PDR.

### Data collection

Data were collected from February 4–27, 2021, through a self-administered online questionnaire at all study sites, with the assistance of local supervisors. To obtain the data for this study, a closed-ended online questionnaire was developed in English and translated into the local languages of Lao PDR (Lao), Thailand (Thai), and Japan (Japanese). To ensure accuracy and limit information loss during translation, backward translation into English was implemented.

### Recruitment method

We recruited undergraduate university students enrolled in health-related programs from the first to the sixth year from three universities: the University of Health Science, Vientiane Capital, Lao PDR; Walailak University, Nakhon Si Thammarat Province, Thailand; and Nagasaki University, Nagasaki City, Japan. Research collaborators sent online links to all undergraduate students from those three universities, using a non-probability convenience sampling method. All participants must read the information sheet and consent online to agree or deny participation before completing the questionnaire. Sample size was identified before starting the questionnaire. We have calculated the sample size based on youth who experienced mental health condition (20%) [10]. The minimum requirement for our samples was 246 people to get enough power of analysis.

### Procedure/tools

Our questionnaire was adapted from the standard questionnaire that can help maintain reliability and consistency in answering the questionnaire from different countries. In this study, we have separated the question into three sections. In Sect. 1, the demographic and academic variables included age, sex, current study status, faculty, study level, history of living with a COVID-19-infected person, frequency of going out, mental health history, and current mental problems. The second section used three standard scales to assess stress, depression, and anxiety: Perceived Stress Scale (PSS-10), Patient Health Questionnaire-9 (PHQ-9), and Generalized Anxiety Disorder Questionnaire-7 (GAD-7), details in Supplement 1 Questionnaires (Sect. 2). The third section collected COVID-19-related variables, including a level of fear of COVID-19, belief in the protective effect of the COVID-19 vaccine, and vaccine hesitancy, details in Supplement 1 Questionnaires (Sect. 3). All questionnaires were completed by the collaborators and cross-checked by local supervisors. Based on the feedback, the tools were reviewed, and some questions were rephrased and rearranged for better flow.

### Statistical analysis

Data was extracted from the online platform into Excel and subsequently prepared for analysis using STATA version 17. Descriptive statistics, including percentages, were used to summarize demographic characteristics and relevant data. The association between each variable was evaluated using Pearson’s chi-square, ${\chi }^{2}$. A higher chi-square value with *p* value < 0.05 means the relationship is statistically significant. To explore predictors of COVID-19 vaccine hesitancy (compare between reported hesitancy group (hesitant or unsure) and no hesitancy group (no hesitation)) [11] across Laos, Thailand, and Japan, logistic regression analysis was conducted to identify significant factors influencing hesitancy using a *p* value < 0.05 with a 95% confidence interval. The crude odds ratio (cOR) corresponds to the association between each predictor and COVID-19 vaccine hesitancy, calculated using simple logistic regression. The adjusted odds ratio (aOR) reflects the multiple logistic regression, where the analysis accounts for participants' characteristics and other relevant covariates to control for potential confounding factors. The adjusted model gives a more robust independent effect from each predictor on vaccine hesitancy.

## Results

### Characteristics of study participants

In the study, 841 participants from three countries [Japan (n = 188), Laos (n = 132), and Thailand (n = 521)] were included (Table 1). The majority of the participants fell within the 18–24 age range. Across all countries, females constitute the primary group compared to males. In Japan, most students (82.45%) took hybrid classes; however, in Laos and Thailand, 78.03% and 87.72% of students took entirely online courses, respectively. Most students had no history of living with COVID-19-infected person (94.77%). In terms of isolation, we checked the frequency of outings and found no significant differences across the three countries, with an average of 87.75% of students going out less than three times a week.

**Table 1:** Characteristics of study participants (*N* = 841)

Characteristic	Total (%)	Japan (%)	Laos (%)	Thailand (%)
1. Age (years)				
18–24	799 (95.12)	167 (88.83)	116 (87.88)	516 (99.04)
25–30	31 (3.69)	16 (8.51)	11 (8.33)	4 (0.77)
≥ 31	11 (1.19)	5 (2.66)	5 (3.79)	1 (0.19)
Total	841 (100.00)	188 (100.00)	132 (100.00)	521 (100.00)
2. Sex				
Male	207 (24.61)	59 (31.38)	49 (37.12)	99 (19.00)
Female	633 (75.27)	129 (68.62)	82 (62.12)	422 (81.00)
Others (missing data)	1 (0.12)	0 (0.00)	1 (0.76)	0 (0.00)
Total	841 (100.00)	188 (100.00)	132 (100.00)	521 (100.00)
3. Current study status				
Entire online class	568 (67.54)	8 (4.26)	103 (78.03)	457 (87.72)
Entire face-to-face class without social distancing	23 (2.73)	4 (2.13)	0 (0.00)	19 (3.65)
Entire face-to-face class with social distancing	40 (4.76)	17 (9.04)	0 (0.00)	23 (4.41)
Combine (face-to-face and online)	176 (20.93)	155 (82.45)	2 (1.52)	19 (3.65)
Close/temporary close	34 (4.04)	4 (2.13)	27 (20.45)	3 (0.58)
Total	841 (100.00)	188 (100.00)	132 (100.00)	521 (100.00)
4. Faculty				
Public health	78 (9.27)	0 (0.00)	0 (0.00)	78 (14.97)
Dentistry	24 (2.85)	24 (12.77)	0 (0.00)	0 (0.00)
Medicine	365 (43.40)	77 (40.96)	131 (99.24)	157 (30.13)
Pharmacy/pharmaceutical science	220 (26.16)	53 (28.19)	1 (0.76)	166 (31.86)
Nurse/nurse assistance	101 (12.01)	34 (18.09)	0 (0.00)	67 (12.86)
Health administration	11 (1.31)	0 (0.00)	0 (0.00)	11 (2.11)
Laboratory sciences	42 (4.99)	0 (0.00)	0 (0.00)	42 (8.06)
Total	841 (100.00)	188 (100.00)	132 (100.00)	521 (100.00)
5. Study level				
Year 1	267 (31.75)	41 (21.81)	34 (25.76)	192 (36.85)
Year 2	157 (18.67)	44 (23.40)	24 (18.18)	89 (17.08)
Year 3	138 (16.41)	28 (14.89)	47 (35.61)	63 (12.09)
Year 4	188 (22.35)	43 (22.87)	13 (9.85)	132 (25.34)
Year 5	46 (5.47)	18 (9.57)	14 (10.61)	14 (2.69)
Year 6	45 (5.35)	14 (7.45)	0 (0.00)	31 (5.95)
Total	841 (100.00)	188 (100.00)	132 (100.00)	521 (100.00)
6. History of living with a COVID-19-infected person				
Yes	42 (4.99)	6 (3.19)	8 (6.06)	28 (5.37)
No	797 (94.77)	180 (95.74)	124 (93.94)	493 (94.63)
Others (missing data)	2 (0.24)	2 (1.06)	0 (0.00)	0 (0.00)
Total	841 (100.00)	188 (100.00)	132 (100.00)	521 (100.00)
7. Frequency of going out per week				
≤ 3 times	738 (87.75)	162 (86.17)	100 (75.76)	476 (91.36)
Between 4 to 6 times	64 (7.61)	18 (9.57)	15 (11.36)	31 (5.95)
≥ 7 times	39 (4.64)	8 (4.26)	17 (12.88)	14 (2.69)
Total	841 (100.00)	188 (100.00)	132 (100.00)	521 (100.00)
8. Students with reported PRIOR mental health problems (mental health history)				
Yes	57 (6.78)	2 (1.06)	6 (4.55)	49 (9.40)
No	783 (93.10)	185 (98.40)	126 (95.45)	472 (90.60)
Others (missing data)	1 (0.12)	1 (0.53)	0 (0.00)	0 (0.00)
Total	841 (100.00)	188 (100.00)	132 (100.00)	521 (100.00)
9. Students with reported CURRENT mental health problems (current mental problems)				
Yes	87 (10.34)	9 (4.79)	18 (13.64)	60 (11.52)
No	754 (89.66)	179 (95.21)	114 (86.36)	461 (88.48)
Total	841 (100.00)	188 (100.00)	132 (100.00)	521 (100.00)

### Self-report on mental-related problems from students

We used the PSS-10, PHQ-9, and GAD-7 scales to evaluate students’ mental status, which are reported in Table 2 and S1–S3. We assess the association between self-reported mental history and mental health scores (PSS-10, PHQ-9, and GAD-7) using Pearson’s chi-squared test (Table 2). Each mental health score in Tables S1–S3 was divided into two groups to determine lower and higher mental health problems by scoring from the standard questionnaires. The result showed an association between both people with prior mental health problems and current mental health problems and all mental health scores (PSS-10, PHQ-9, and GAD-7). We additionally identify the association between mental health score with sex and country (Table S4). The results showed that each mental health score tended to be higher for women than for men, but only the PSS-10 score was statistically significantly associated with gender. Concerning the differences between the countries, Thailand is the country with the highest proportion of respondents answering “High” and “Very High” on the PSS-10 (Table S1), indicating that they are exposed to high levels of stress. Japan has the highest proportion (56.91%) of “None” for depression in the PHQ-9, and the proportion of people with severe depression is also low (Table S2), indicating a low propensity to depression. The GAD-7 scores reported anxiety levels among the students. In terms of anxiety, as measured by the GAD-7, Laos had the highest anxiety score (Table S3). We observed country-specific characteristics for stress, depression, and anxiety components.

**Table 2:** Association between self-reported prior mental health, current mental health status, sex, country with mental health score (PSS-10, PHQ-9, GAD-7)

PSS-10	Very low (*N*, %)		Low to very high (*N*, %)		Total	χ^2^	*df*	*p* value
Students with reported PRIOR mental health problems (*N* = 840)^a^								
With PRIOR mental health problems	181 (96.79%)		602 (92.19%)		783	4.87	1	< 0.05*
Without PRIOR mental health problems	6 (3.21%)		51 (7.81%)		57			
Total	187 (100.00%)		653 (100.00%)		840			
Students with reported CURRENT mental health problems (*N* = 841)								
With CURRENT mental health problems	181 (96.79%)		573 (87.61%)		754	13.20	1	< 0.05*
Without CURRENT mental health problems	6 (3.21%)		81 (12.39%)		87			
Total	187 (100.00%)		654 (100.00%)		841			
Sex (*N* = 840)^a^								
Male	62 (33.16%)		145 (22.21%)		207	9.39	1	< 0.05*
Female	125 (66.84%)		508 (77.79%)		633			
Total	187 (100.00%)		653 (100.00%)		840			

PHQ-9	None (*N*, %)		Mild to severe (*N*, %)		Total	χ^2^	*df*	*p* value
Students with reported PRIOR mental health problems (*N* = 840)^a^								
With PRIOR mental health problems	367 (98.39%)		416 (89.08%)		783	28.43	1	< 0.05*
Without PRIOR mental health problems	6 (1.61%)		51 (10.92%)		57			
Total	373		467		840			
Students with reported CURRENT mental health problems (*N* = 841)								
With CURRENT mental health problems	362 (97.05%)		392 (83.76%)		754	39.53	1	< 0.05*
Without CURRENT mental health problems	11 (2.95%)		76 (16.24%)		87			
Total	373 (100.00%)		468 (100.00%)		841			
Sex (*N* = 840)^a^								
Male	97 (26.01%)		110 (23.55%)		207	0.67	1	0.41
Female	276 (73.99%)		357 (76.45%)		633			
Total	373 (100.00%)		467 (100.00%)		840			

GAD-7	Minimal (*N*, %)		Mild to severe (*N*, %)		Total	χ^2^	*df*	*p* value
Students with reported PRIOR mental health problems (*N* = 840)^a^								
With PRIOR mental health problems	463 (96.86%)		320 (88.40%)		783	23.33	1	< 0.05*
Without PRIOR mental health problems	15 (3.14%)		42 (11.60%)		57			
Total	478 (100.00%)		362 (100.00%)		840			
Students with reported CURRENT mental health problems (*N* = 841)								
With CURRENT mental health problems	458 (95.62%)		296 (81.77%)		754	42.63	1	< 0.05*
Without CURRENT mental health problems	21 (4.38%)		66 (18.23%)		87			
Total	479 (100.00%)		362 (100.00%)		841			
Sex (*N* = 840)^a^								
Male	128 (26.72)		79 (21.88)		207	2.60	1	0.11
Female	351 (73.28)		282 (78.12)		633			
Total	479 (100.00%)		361 (100.00%)		840			

*Significant at *p* < 0.05 (${\chi }^{2}$ = Pearson’s chi-squared); degree of freedom (*df*) Perceived Stress Scale (PSS-10); Patient Health Questionnaire-9 (PHQ-9); Generalized Anxiety Disorder-7 (GAD-7); for more details on S1-S3 (supplement section)

**“Sex” and “Students with reported PRIOR mental health problems” variables had one missing participant

### Self-reported COVID-19 vaccine hesitancy

**Fig. 1:**
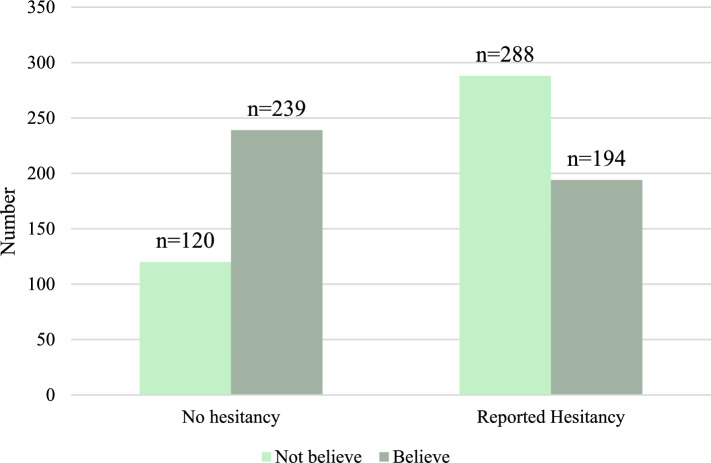
COVID-19 vaccine hesitancy and students’ belief in the protection of COVID-19 vaccines

Data on COVID-19 vaccine hesitancy were collected based on self-reports. We collected data on COVID-19 vaccine hesitancy with response options: "yes", "no", and "unsure". For analysis purposes, individuals who responded "yes" or "unsure" were grouped as the reported hesitancy group. Table 3 presents the distribution of vaccine hesitancy among respondents from Thailand, Japan, and Laos. Among the respondents, “reported hesitancy” was highest in Thailand (71.40%), followed by Laos (48.48%) and Japan (24.47%). These results indicated that Japan had the lowest vaccine hesitancy, significantly different from Thailand and Laos. To explore what might account for this significant difference, an additional question was asked to assess confidence in the vaccine's efficacy: "Do you believe that COVID-19 vaccines can protect you from serious diseases?". As expected, the proportion of students who believed in the vaccine's efficacy was highest in the “no hesitancy” group (Fig. 1).

**Table 3:** Association between COVID-19 vaccine hesitancy report in each country

COVID-19 vaccine hesitancy	Country			Comparison between country, ${\upchi }^{2}$ (*p* value*)*		
	Japan, *N* (%)	Laos, *N* (%)	Thailand, *N* (%)	Japan vs. Thailand	Japan vs. Laos	Thailand vs. Laos
No hesitancy	142 (75.53)	68 (51.52)	149 (28.60)	${\upchi }^{2}$=125.76 (*p* < 0.05)	${\upchi }^{2}$=19.83 (*p* < 0.05)	${\upchi }^{2}$=24.93 (*p* < 0.05)
Reported hesitancy	46 (24.47)	64 (48.48)	372 (71.40)			
Total	521(100.00)	188 (100.00)	132 (100.00)			

*Significant at *p* < 0.05; (${\chi }^{2}$ = Pearson’s Chi-squared)

### Association between participant’s characteristics and reported COVID-19 vaccine hesitancy

Several factors identified were associated with COVID-19 vaccine hesitancy and participant’s characteristics (Tables S5, S6). Females reported being more hesitant about the COVID-19 vaccine compared with males (63.82%, female vs. 37.20%, male, Table 4). The results revealed that being female was associated with almost double the odds of being hesitant compared to being a male (aOR 2.43, 95% CI 1.68–3.51). There was a report on vaccine hesitancy in all three countries. However, because Japan revealed minor hesitancy compared to Laos and Thailand, we used Japan as the reference category for the analysis (Table 4). It demonstrates that Lao students were almost twice as likely to hesitate to take the COVID-19 vaccines than the Japanese (aOR 1.97, 95%CI 1.15–3.35). Thailand participants reported the highest level of hesitancy, which was more than five times higher than Japan’s (aOR 5.96, 95%CI 3.97–8.95).

**Table 4:** Logistic regression between participants' characteristics and vaccine hesitancy

Other factors		Hesitation to COVID-19 vaccine			Crude odds ratio			Adjusted odds ratio		*p* value
		No hesitancy		Reported hesitancy						
Gender^a^										
Male		130 (62.80)		77 (37.20)	1.00 (as reference)			1.00 (as reference)		
Female		229 (36.18)		404 (63.82)	2.98 (2.15–4.12)			2.43 (1.68–3.51)		< 0.05*
Country										
Japan		142 (75.53)		46 (24.47)	1.00 (as reference)			1.00 (as reference)		
Lao		68 (51.52)		64 (48.48)	2.91(1.80–4.68)			1.97 (1.15–3.35)		0.01*
Thailand		149 (28.60)		372 (71.40)	7.71 (5.25–11.31)			5.96 (3.97–8.95)		< 0.05*
Living with COVID-19 patients										
Without COVID-19 patients		342 (42.91)		455 (57.09)	1.00 (as reference)			1.00 (as reference)		
With COVID-19 patients		16 (38.10)		26 (61.90)	1.22 (0.65–2.31)			1.13 (0.52–2.45)		0.76
Others (missing data)		1 (50.00)		1 (50.00)	–			–		
Fear of COVID-19										
Low (1–4)		54 (64.29)		30 (35.71)	1.00 (as reference)			1.00 (as reference)		
Moderate (5–7)		149 (47.45)		165 (52.55)	1.99 (1.21–3.28)			1.50 (0.82–2.76)		0.19
High (8–10)		156 (35.21)		287 (64.79)	3.31 (2.03–5.39)			1.93 (1.06–3.50)		0.03*
Belief in COVID-19 vaccine protection										
Yes		239 (55.20)		194 (44.80)	1.00 (as reference)			1.00 (as reference)		
No		120 (29.41)		288 (70.59)	2.96 (2.22–3.93)			2.59 (1.86–3.59)		< 0.05*

^a^One missing data

*Significant at *p* < 0.05 for adjusted odds ratio, 95% confidence interval (95% CI)

The different levels of fear toward COVID-19 are illustrated in Table S10. The scale is scored from 1 (lowest) to 10 (highest). The subgroups were identified as 1–4 (low), 5–7 (moderate), and 8–10 (high) fear. Thailand and Laos reported the highest proportion of fear among the “reported hesitancy” group, whereas Japan’s fear level was lower. Those who scored higher (score 8–10) on the fear scale were likely to hesitate to receive the vaccine (aOR 1.93, 95% CI 1.06–3.50, Table 4). The students who did not believe in the vaccine’s effectiveness had two times higher odds of hesitating to receive the COVID-19 vaccine (aOR 2.59, 95% CI 1.86–3.59, Table 4).

### Association between mental health scores and the COVID-19 vaccine hesitancy

We analyzed the association between mental health scores (PSS-10, PHQ-9, and GAD-7) and COVID-19 vaccine hesitancy (Table 5). All three scores demonstrated the same trend. Individuals with higher mental health scores were more likely to exhibit vaccine hesitancy for COVID-19. Especially, the higher the stress score, the greater the hesitancy to receive the COVID-19 vaccine. In the “very high” stress group (PSS-10 score: 21–40), aOR was 2.67 (95% CI 1.45–4.93), indicating a statistically significant effect of high stress levels on vaccine hesitancy. For depression as assessed by the PHQ-9 and anxiety as assessed by the GAD-7, people with higher levels of depression or anxiety tended to be more hesitant to receive the COVID-19 vaccine, although there were few statistically significant associations with these two scores.

**Table 5:** Logistic regression of mental-related problems to COVID-19 vaccine hesitancy

Mental health score	Hesitation to COVID-19 vaccine		Crude odds ratio	Adjusted odds ratio	*p* value
	No hesitancy	Reported hesitancy			
Perceived Stress Scale (PSS-10)					
Very low (score 0–7)	92 (49.20)	95 (50.80)	1.00 (as reference)		
Low (score 8–11)	85 (54.49)	71 (45.51)	0.81 (0.53–1.24)	0.82 (0.50–1.34)	0.43
Average (score 12–15)	85 (45.21)	103 (54.79)	1.17 (0.78–1.76)	1.32 (0.82–2.12)	0.26
High (score 16–20)	77 (35.65)	139 (64.35)	1.75 (1.17–2.61)	1.55 (0.98–2.46)	0.06
Very high (score 21–40)	20 (21.28)	74 (78.72)	3.58 (2.02–6.35)	2.67 (1.45–4.93)	< 0.05*
Patient Health Questionnaire-9 (PHQ-9)					
None to minimal (score 0–4)	186 (49.87)	187 (50.13)	1.00 (as reference)		
Mild (score 5–9)	95 (37.55)	158 (62.45)	1.65 (1.19–2.29)	1.64 (1.13–2.39)	0.01*
Moderate (score 10–14)	43 (38.74)	68 (61.26)	1.57 (1.02–2.43)	1.45 (0.91–2.30)	0.12
Moderate severe (score 15–19)	23 (35.38)	42 (64.62)	1.82 (1.05–3.14)	1.47 (0.78–2.77)	0.23
Severe (score 20–27)	12 (30.77)	27 (69.23)	2.24 (1.10–4.55)	2.28 (1.02–5.12)	< 0.05*
Generalized Anxiety Disorder-7 (GAD-7)					
Minimal (score 0–4)	232 (48.43)	247 (51.57)	1.00 (as reference)		
Mild (score 5–9)	76 (36.54)	132 (63.46)	1.63 (1.17–2.28)	1.87 (1.28–2.74)	< 0.05*
Moderate (score 10–14)	38 (35.19)	70 (64.81)	1.73 (1.12–2.67)	1.38 (0.85–2.26)	0.20
Severe (score ≥ 15)	13 (28.26)	33 (71.74)	2.38 (1.22–4.64)	1.95 (0.92–4.12)	0.08

*Significant at *p* < 0.05 for adjusted odds ratio, 95% confidence interval (95% CI)

## Discussion

The COVID-19 pandemic had a significant impact on mental health, increasing depression, anxiety, and stress. WHO reported a 25% rise in the global prevalence of anxiety and depression worldwide in 2020 [12]. Before the COVID-19 pandemic, mental health challenges among adolescents and young adults were already a significant global concern [13–15], while this problem may affect the uptake of COVID-19 vaccine as well.

We investigated the association between the mental health of health care-related students and their COVID-19 vaccine hesitancy. The WHO defines vaccine hesitancy as a “delay in the acceptance of vaccines, despite the availability of vaccination services” [16]. The level of hesitancy ranges from complete acceptance to refusal despite the availability of the vaccine. New vaccines are usually accompanied by uncertainties owing to unknown side effects, making target groups skeptical about whether the intervention would work or worsen their health conditions and triggering hesitancy [17].

This study demonstrated that the mental health could affect to decision to get the COVID-19 vaccine. We found that students with very high stress levels in PSS-10 were more likely to refuse COVID-19 vaccines and to score higher on depression (PHQ-9) and anxiety (GAD-7) (Table 5). These findings are consistent with the study by Sekizawa et al., which showed that mental health conditions such as depression and generalized anxiety were associated with vaccine hesitancy [18]. Fear-related hesitancy was reflected in this study, as those with a higher score for fear of COVID-19 were likely to hesitate to receive the vaccine (Table S10). This finding concurred with a study by Yeşiltepe et al.[19], which revealed that the inconsistency in evidence regarding the effectiveness of the vaccines against COVID-19 and related side effects for the vaccines already in the market increased doubts [19].

However, recent research has shown that unspecific anxiety and depressive symptoms were not significantly associated with vaccine acceptance [20], which does not align with our analysis. As research on this topic has yielded inconsistent results, further research is needed to discuss the relationship between mental health status and vaccine acceptance.

This study illustrated that Thai and Laotian students were more worried about receiving COVID-19 vaccines than Japanese students (Table 3). This result is similar to the previous survey among Thai parents and guardians [21]. One reason for this is government policy, which may affect vaccine hesitancy. Previous research has demonstrated that trust in government policy and confidence in effectiveness from vaccine is significantly lower compared to the non-hesitant group [22]. Our result also showed that students who believed in protecting against COVID-19 vaccines had lower vaccine hesitance (Fig. 1). However, the underlying reasons for the heightened concern among Thai and Laotian students regarding COVID-19 vaccines remain unclear and require further investigation. These regional differences highlight the need for tailored public health strategies to address vaccine hesitancy effectively.

We demonstrated that female students exhibited greater hesitancy than males (Table 4). Previous studies have indicated a similar trend among the general population, showing that women have higher odds of COVID-19 vaccine hesitancy than men (OR = 1.52; p < 0.05) [23]. A study from Japan reported that younger populations, particularly females, are highly likely to be hesitant to receive COVID-19 vaccines [24]. It is widely recognized that young people are at low risk of severe disease from COVID-19. Therefore, when students weigh the benefits against the side effects of vaccines, they should be more concerned about the side effects and tend to avoid vaccination. It is generally said that women tend to be more proactive in health-seeking behavior. This could positively affect improving vaccination rates; however, many studies, including ours, have found the opposite effect that women hesitate more than men. The results of mental health status in our research showed that female students had higher levels of anxiety than male students. We think that when fear of developing disease increases, it promotes health-seeking behavior, but when fears of medical services themselves increase, it accelerates a strong sense of avoidance. Vaccines against COVID-19, especially the RNA vaccine, have been developed rapidly despite some concerns from the public. The fact that women showed a more conservative attitude toward a novel vaccine can be explained by the psychological characteristics of women.

An additional study provided further information, highlighting that sex and socioeconomic status play complex roles in shaping people’s vaccine hesitancy. Specifically, women living in poverty or currently employed are hesitant about vaccines [25]. Taking all this into consideration, vaccine hesitancy is not simply related to gender, and we need to be aware of gender-related gaps, such as mental health status and socioeconomic status.

Our research subsequently identified factors related to the mental health status among healthcare-related undergraduate students. Female gender is factors that reported as factor similar to other studies [15, 26]. Cross-country difference was an additional factor influencing mental health outcomes especially Thai students were more likely to report high levels of mental health issues during the pandemic which comparable to another research [27]. However, no similar survey has ever been conducted in Laos. The social distance and the lockdown interventions against COVID-19 led to social isolation in the general population worsened mental health since the pandemic especially some preexisting mental health problems [28–30]. During our survey period, the lockdown strategy was used in Thailand and Laos may affect higher mental health [31], though the restrictions on the lockdown policy were less strict in Japan [32]. In this study, students with a history of prior mental health problems reported a higher risk of mental health and higher scores than students without mental health problems (Table 2). Unfortunately, due to the limitations of our cross-sectional study design, we could not compare the results of individuals before and after the pandemic.

Taking together with our findings and previous studies, it may be necessary to prioritize early prevention and intervention programs for students without a history of mental health problems, to address the increased psychological distress caused by social isolation. In addition, ongoing support should be provided for students with existing mental health needs, as mental health problems may contribute to hesitation toward receiving the COVID-19 vaccine.

### Limitations of this study

This study examined vaccine hesitancy and mental health during COVID-19 compared with samples from Southeast Asian countries, including Japan. However, we still have some limitations as follows. This study included participants from health science programs, which may limit the generalizability of the findings to the broader university student population. Furthermore, as data were collected from universities in Asian countries, the results may not be generalizable to students in other regions. To establish the causal relationships between vaccine hesitancy and mental health may need to be confirmed. Because, at that time of survey is cross-sectional, decisions regarding COVID-19 vaccination may have been shaped by unmeasurable factors, such as the vaccine development, trust in vaccine manufacturers, and the local outbreaks, although we have tried to manage some confounding measures. This study may have a relatively low response rate due to the recruitment procedure. This limitation is likely attributable to the timing of data collection, which occurred during the COVID-19 pandemic. Moreover, an online data collection method may have limited participation among students with inadequate internet access and distribution channels. Despite these challenges, our final sample size provided sufficient statistical power to produce results consistently compared to previous studies [5–8, 33]. Finally, the participants number varied across countries, with a larger proportion from Thailand. The unequal distribution of participants may have influenced the comparative analysis. However, our questionnaire was used or adapted from standardized instruments, which helps ensure consistency of responses across different countries.

## Conclusion

Poor mental health might contribute to COVID-19 vaccine hesitancy among healthcare-related undergraduate student especially female gender. Interventions, including tailored support, awareness campaigns, and psychological services, can foster trust and vaccine uptake.

### Further investigation

Implementation to improve COVID-19 vaccine uptake will be more challenging in groups with underlying conditions, especially mental illness. External factors in each country may also affect vaccine uptake. Future research should collect data from multiple timepoints, including after the pandemic, to confirm our results to develop effective future interventions. To enhance the generalizability of our findings, it is necessary to broaden the scope of this study by including a more comprehensive range of participants from other developing countries and expand to other student populations.

## Supplementary Information

Supplementary Material 1.

## Data Availability

No data sets were generated or analysed during the current study.

## Abbreviations

COVIS-19: Coronavirus disease 2019
GAD-7: Generalized anxiety disorder-7
PSS-10: Perceived Stress Scale
PHQ-9: Patient Health Questionnaire-9
