# Supplementary material for: Mental health and COVID-19 vaccine hesitancy among health-related university students: a cross-sectional multi-center study
Source: Trop Med Health. 2025 May 19;53:71. doi: 10.1186/s41182-025-00751-3 (PMC12087161; doi:10.1186/s41182-025-00751-3)
Supplement: Supplementary file 1 — Supplementary Material 1. [file 41182_2025_751_MOESM1_ESM.docx]

**Supplements**

**Table S1** Perceived Stress Scale (PSS-10) score for three different Asian countries

| **Country** | **Stress level** | | | | | **Total** |
| --- | --- | --- | --- | --- | --- | --- |
|  | **Very low** | **Low** | **Average** | **High** | **Very high** |  |
| Japan | 39  (20.74%) | 46  (24.47%) | 54  (28.72%) | 41  (21.81%) | 8  (4.26%) | 188  (100%) |
| Laos | 42  (31.82%) | 23  (17.42%) | 28  (21.21%) | 27  (20.45%) | 12  (9.09%) | 132  (100%) |
| Thailand | 106 (20.35%) | 87  (16.7%) | 106 (20.35%) | 148  (28.41 %) | 74  (14.2%) | 521  (100%) |
| Total | 187 (22.24%) | 156 (18.55%) | 188 (22.35%) | 216 (25.68%) | 94  (11.18%) | 841  (100%) |

Note. Health concern interpretation was as follows: 0–7 (very low), 8–11 (low), 12–15 (average), 16–20 (high), and 21–40 (very high).

**Table S2** Depression levels using the PHQ-9 score for three different Asian countries.

| **Country** | **Depression level** | | | | | **Total**  (100%) |
| --- | --- | --- | --- | --- | --- | --- |
|  | **None** | **Mild** | **Moderate** | **Moderate to severe** | **Severe** |  |
| Japan | 107 (56.91%) | 48  (25.53%) | 19  (10.11%) | 9  (4.79%) | 5  (2.66%) | 188 |
| Laos | 47  (35.61%) | 35  (26.52%) | 20  (15.15%) | 17  (12.88%) | 13  (9.85%) | 132 |
| Thailand | 219 (42.03%) | 170 (32.63%) | 72  (13.82%) | 39  (7.49%) | 21  (4.03%) | 521 |
| Total | 373 (44.35%) | 253 (30.08%) | 111  (13.2%) | 65  (7.73%) | 39  (4.64%) | 841 |

Note. PHQ-9 interpretation: 0–4 none to minimal, 5–9 (mild), 10–14 (moderate), 15–19 (moderate to severe), and 20–27 (severe).

**Table S3** Anxiety levels using the GAD-7 score for three different Asian countries.

| **Country** | **Anxiety level** | | | | **Total** |
| --- | --- | --- | --- | --- | --- |
|  | **Minimal** | **Mild** | **Moderate** | **Severe** |  |
| Japan | 125  (66.49%) | 46  (24.47%) | 13  (6.91%) | 4  (2.13%) | 188  (100%) |
| Laos | 54  (40.91%) | 40  (30.30%) | 21  (15.91%) | 17  (12.88%) | 132  (100%) |
| Thailand | 300  (57.58%) | 122  (23.42%) | 74  (14.2%) | 25  (4.8%) | 521  (100%) |
| Total | 479  (56.96%) | 208  (24.73%) | 108  (12.84%) | 46  (5.47%) | 841  (100%) |

Note. GAD-7 scoring: 0–4 (minimal anxiety), 5–9 (mild anxiety), 10–14 (moderate anxiety), and greater than 15 (severe anxiety).

**Table S4** Sub-group between reported mental health status by sex and country

| Subgroup | Reported as prior mental health problem | | | Reported as a current mental health problem | | |
| --- | --- | --- | --- | --- | --- | --- |
|  | No,  N (%) | Yes,  N (%) | *p-value* | No,  N (%) | Yes,  N (%) | *p-value* |
| **Subgroup by sex*** | | | | | | |
| Male | 193 (93.69) | 13 (6.31) | $\chi^{2}$ = 0.06 (p=0.81) | 190 (91.79) | 17 ( 8.21) | $\chi^{2}$ = 1.23  (p= 0.27) |
| Female | 590 (93.21) | 43 (6.79) |  | 564 (89.10) | 69 (10.90) |  |
| **Subgroup by country** | | | | | | |
| Japan | 185 (98.93) | 2 (1.07) | $\chi^{2}$ = 16.36 (p<0.05*) | 179 (95.21)  114 (86.36)  461 (88.48) | 9 (4.79) | $\chi^{2}$ = 8.57  (p= 0.01*) |
| Laos | 126 (95.45) | 6 (4.55) |  |  | 18 (13.64) |  |
| Thailand | 472 (90.60) | 49 (9.40) |  |  | 60 (11.52) |  |

Note. *Significant at *p*<0.05*; ($\chi^{2}$ = Pearson’s chi-squared); N = number

**Table S5** Association between participants' characteristics with vaccine hesitancy (Simple logistic regression)

| **Other factors** | **Crude**  **Odds ratio** | **SE** | **z** | ***p-value*** | **95% CI** |
| --- | --- | --- | --- | --- | --- |
| **Gender** | | | | | |
| Male | 1.00 (as reference) | | | | |
| Female | 2.98 | 0.49 | 6.58 | <0.05* | 2.15-4.12 |
| **Country** | | | | | |
| Japan | 1.00 (as reference) | | | | |
| Lao | 2.91 | 0.71 | 4.38 | <0.05*** | 1.80-4.68 |
| Thailand | 7.71 | 1.51 | 10.44 | <0.05*** | 5.25-11.31 |
| **Living with COVID-19 patients** | | | | | |
| Without COVID-19 patients | 1.00 (as reference) | | | | |
| With COVID-19 patients | 1.22 | 0.40 | 0.61 | 0.54 | 0.65-2.31 |
| **Fear of COVID-19** | | | | | |
| Low (1–4) | 1.00 (as reference) | | | | |
| Moderate (5–7) | 1.99 | 0.51 | 2.71 | <0.05*** | 1.21-3.28 |
| High (8–10) | 3.31 | 0.82 | 4.82 | <0.05*** | 2.03-5.39 |
| **Belief in COVID-19 vaccine protection** | | | | | |
| Yes | 1.00 (as reference) | | | | |
| No | 2.96 | 0.43 | 7.45 | <0.05*** | 2.22-3.93 |

Note. *Significant at *p-value*<0.05 (crude odds ratio), 95% confidence interval (95% CI)

**Table S6** Association between participants' characteristics with vaccine hesitancy (Multiple logistic regression)

| Other factors | Adjusted  Odds ratio | SE | z | *p-value* | 95% CI |
| --- | --- | --- | --- | --- | --- |
| Gender | | | | | |
| Male | 1.00 (as reference) | | | | |
| Female | 2.43 | 0.46 | 4.72 | <0.05* | 1.68-3.51 |
| Country | | | | | |
| Japan | 1.00 (as reference) | | | | |
| Lao | 1.97 | 0.53 | 2.49 | 0.01* | 1.15-3.35 |
| Thailand | 5.96 | 1.24 | 8.60 | <0.05* | 3.97-8.95 |
| Living with COVID-19 patients | | | | | |
| With COVID-19 patients | 1.00 (as reference) | | | | |
| Without COVID-19 patients | 1.13 | 0.45 | 0.30 | 0.76 | 0.52-2.45 |
| Fear of COVID-19 | | | | | |
| Low (1–4) | 1.00 (as reference) | | | | |
| Moderate (5–7) | 1.50 | 0.47 | 1.30 | 0.19 | 0.82-2.76 |
| High (8–10) | 1.93 | 0.59 | 2.16 | 0.03* | 1.06-3.50 |
| Belief in COVID-19 vaccine protection | | | | | |
| Yes | 1.00 (as reference) | | | | |
| No | 2.59 | 0.43 | 5.68 | <0.05* | 1.86-3.59 |

Note. *Significant at *p-value*<0.05 (adjusted odds ratio), 95% confidence interval (95% CI)

**Table S7** Mental-related scores to COVID-19 vaccine hesitancy (simple logistic regression)

| Mental health score | Crude  Odds ratio | SE | z | *p-value* | 95% CI |
| --- | --- | --- | --- | --- | --- |
| Perceived Stress Scale (PSS-10) | | | | | |
| Very low (score 0–7) | 1.00 (as reference) | | | | |
| Low (score 8–11) | 0.81 | 0.18 | -0.98 | 0.33 | 0.53-1.24 |
| Average (score 12–15) | 1.17 | 0.24 | 0.77 | 0.44 | 0.78-1.76 |
| High (score 16–20) | 1.75 | 0.36 | 2.74 | 0.01* | 1.17-2.61 |
| Very high (score 21–40) | 3.58 | 1.05 | 4.38 | <0.05* | 2.02-6.35 |
| Patient Health Questionnaire-9 (PHQ-9) | | | | | |
| None to minimal (score 0–4) | 1.00 (as reference) | | | | |
| Mild (score 5–9) | 1.65 | 0.28 | 3.03 | <0.05* | 1.19-2.29 |
| Moderate (score 10–14) | 1.57 | 0.35 | 2.05 | 0.04* | 1.02-2.43 |
| Moderate severe (score 15–19) | 1.82 | 0.51 | 2.14 | 0.03* | 1.05-3.14 |
| Severe (score 20–27) | 2.24 | 0.81 | 2.22 | 0.03* | 1.10-4.55 |
| Generalized Anxiety Disorder-7 (GAD-7) | | | | | |
| Minimal (score 0–4) | 1.00 (as reference) | | | | |
| Mild (score 5–9) | 1.63 | 0.28 | 2.87 | <0.05* | 1.17-2.28 |
| Moderate (score 10–14) | 1.73 | 0.38 | 2.48 | 0.01* | 1.12-2.67 |
| Severe (score >15) | 2.38 | 0.81 | 2.55 | 0.01* | 1.22-4.64 |

Note. *Significant at *p-value*<0.05 (crude odds ratio), 95% confidence interval (95% CI)

**Table S8** Mental-related scores to COVID-19 vaccine hesitancy (multiple logistic regression)

| Mental health score | Adjusted Odds Ratio | SE | z | *p-value* | 95% CI |
| --- | --- | --- | --- | --- | --- |
| Perceived Stress Scale (PSS-10) | | | | | |
| Very low (score 0–7) | 1.00 (as reference) | | | | |
| Low (score 8–11) | 0.82 | 0.21 | -0.80 | 0.43 | 0.50-1.34 |
| Average (score 12–15) | 1.32 | 0.32 | 1.13 | 0.26 | 0.82-2.12 |
| High (score 16–20) | 1.55 | 0.36 | 1.86 | 0.06 | 0.98-2.46 |
| Very high (score 21–40) | 2.67 | 0.83 | 3.15 | <0.05* | 1.45-4.93 |
| Patient Health Questionnaire-9 (PHQ-9) | | | | | |
| None to minimal (score 0–4) | 1.00 (as reference) | | | | |
| Mild (score 5–9) | 1.64 | 0.32 | 2.58 | 0.01* | 1.13-2.39 |
| Moderate (score 10–14) | 1.45 | 0.34 | 1.55 | 0.12 | 0.91-2.30 |
| Moderate severe (score 15–19) | 1.47 | 0.48 | 1.19 | 0.23 | 0.78-2.77 |
| Severe (score 20–27) | 2.28 | 0.94 | 2.00 | <0.05* | 1.02-5.12 |
| Generalized Anxiety Disorder-7 (GAD-7) | | | | | |
| Minimal (score 0–4) | 1.00 (as reference) | | | | |
| Mild (score 5–9) | 1.87 | 0.36 | 3.25 | <0.05* | 1.28-2.74 |
| Moderate (score 10–14) | 1.38 | 0.35 | 1.29 | 0.20 | 0.85-2.26 |
| Severe (score >15) | 1.95 | 0.75 | 1.74 | 0.08 | 0.92-4.12 |

Note. *Significant at *p-value*<0.05 (adjusted odds ratio), 95% confidence interval (95% CI)

**Table S9.** COVID-19 vaccine hesitancy and free time [How often did you travel outside campus during the COVID-19 pandemic per week?]

| **Times/week** | **Vaccine hesitancy, N (%)** | | | **Total** |
| --- | --- | --- | --- | --- |
|  | **No** | **Not sure** | **Yes** |  |
| Japan | | | | |
| 0 | 28 (19.72%) | 4 (36.36%) | 5 (14.29%) | 37 (19.68%) |
| 1 | 44 (30.99%) | 6 (54.55%) | 17 (48.57%) | 67 (35.64%) |
| 2 | 26 (18.31%) | 1 (9.09%) | 6 (17.14%) | 33 (17.55%) |
| 3 | 24 (16.90%) | 0 (0.00%) | 1 (2.86%) | 25 (13.30%) |
| 4 | 5 (3.52%) | 0 (0.00%) | 2 (5.71%) | 7 (3.72%) |
| More than four times | 15 (10.56%) | 0 (0.00%) | 4 (11.43%) | 19 (10.11%) |
| **Total** | **142 (100%)** | **11 (100%)** | **35 (100%)** | **188 (100%)** |
| Laos | | | | |
| 0 | 16 (23.53%) | 1 (11.11%) | 16 (29.09%) | 33 (25%) |
| 1 | 12 (17.65%) | 2 (22.22%) | 9 (16.36%) | 23 (17.42%) |
| 2 | 10 (14.71%) | 4 (44.44%) | 13 (23.64%) | 27 (20.45%) |
| 3 | 13 (19.12%) | 0 (0.00%) | 4 (7.27%) | 17 (12.88%) |
| 4 | 2 (2.94%) | 0 (0.00%) | 2 (3.64%) | 4 (3.03%) |
| More than four times | 15 (22.06%) | 2 (22.22%) | 11 (20.00%) | 28 (21.21%) |
| **Total** | **68 (100%)** | **9 (100%)** | **55 (100%)** | **132 (100%)** |
| Thailand | | | | |
| 0 | 45 (30.2%) | 14 (23.33%) | 98 (31.41%) | 157 (30.13%) |
| 1 | 42 (28.19%) | 26 (43.33%) | 99 (31.73%) | 167 (32.05%) |
| 2 | 24 (16.11%) | 12 (20%) | 65 (20.83%) | 101 (19.39%) |
| 3 | 17 (11.41%) | 2 (3.33%) | 32 (10.26%) | 51 (9.79%) |
| 4 | 8 (5.37%) | 2 (3.33%) | 7 (2.24%) | 17 (3.26%) |
| More than four times | 13 (8.72%) | 4 (6.67%) | 11 (3.53%) | 28 (5.37%) |
| **Total** | 149(100%) | 60 (100%) | 312 (100%) | 521 (100%) |
| **Grand Total** | **359 (100%)** | **80 (100%)** | **402 (100%)** | **841 (100%)** |

**Table S10** Fear of COVID-19 proportion among three countries (percentage).

|  | Level of fear | | | | | |
| --- | --- | --- | --- | --- | --- | --- |
| Hesitancy, N (%) | **Low** | **Moderate** | | **High** | | **Total** |
| Japan |  |  | |  | |  |
| No hesitancy | 33 (82.5) | 57 (71.25) | | 52 (76.47) | | 142 (75.53) |
| Reported hesitancy | 7 (17.5) | 23 (28.75) | | 16 (23.53) | | 46 (24.47) |
| Total | 40 (100.00) | 80 (100.00) | | 68 (100.00) | | 188 (100.00) |
| Laos |  | |  | |  | |
| No hesitancy | 6 (66.67) | 28 (63.64) | | 34 (43.04) | | 68 (51.52) |
| Reported hesitancy | 3 (33.33) | 16 (36.36) | | 45 (56.96) | | 64 (48.48) |
| Total | 9 (100.00) | 44 (100.00) | | 79 (100.00) | | 132 (100.00) |
| Thailand |  |  | |  | |  |
| No hesitancy | 15 (42.86) | 64 (33.68) | | 70 (23.65) | | 149 (28.60) |
| Reported hesitancy | 20 (57.14) | 126 (66.32) | | 226 (76.35) | | 372 (71.40) |
| Total | 35 (100.00) | 190 (100.00) | | 296 (100.00) | | 521 (100.00) |

Note: Fear score: Low (1–4), Moderate (5–7), High (8–10); Reported hesitancy included hesitant and unsure)

**Supplement 1. Questionnaires**

**Section 1 demographic and academic variables**

1. Age:
   - 18–24 years
   - 25–30 years
   - 31 years or more
2. Sex:
   - Male
   - Female
3. Current study status:
   - Entirely online classes
   - Entirely face-to-face classes without social distancing
   - Entirely face-to-face classes with social distancing
   - Combination of face-to-face and online classes
   - Classes are closed or temporarily closed
4. Faculty:
   - Public Health
   - Dentistry
   - Medicine
   - Pharmacy / Pharmaceutical Science
   - Nursing / Nursing Assistance
   - Health Administration
   - Laboratory Sciences
5. Study Level:
   - Year 1
   - Year 2
   - Year 3
   - Year 4
   - Year 5
   - Year 6
6. History of living with a COVID-19-infected person
   - Yes
   - No
7. Frequency of going out per week: we recategorize the data into three groups including: Less than 3 times, between 4 and 6 times, and 7 times or more to make clearer explanations.
   - 0
   - 1
   - 2
   - 3
   - 4
   - More than four times
8. Students with reported PRIOR mental health problems (Mental health history)
   - Yes: Mental condition (anxiety, depression, stress, etc.)
   - No
9. Students with reported CURRENT mental health problems (Current mental problems)
   - Yes: Mental condition (anxiety, depression, stress, etc.)
   - No

**Section 2 Mental related variables**

1. **The Perceived Stress Scale (PSS-10)**

The PSS-10 is a psychological instrument to measure the perception of stress to evaluate how unpredictable, uncontrollable, and overwhelming individuals have found their lives over the past month[1]. It contains 10 items with total scores ranging from 0 to 40; higher scores indicate higher levels of perceived stress. The interpretation was explained in the reference [2]. The standard scale is available in English [1,3], Japanese [4], and Thai [5]. Back-translation was performed in the same manner for Laos.

**The PSS-10 questions**

Over the last 4 weeks, how about your feelings and thoughts?

Scale: Never = 0 Almost Never = 1 Sometimes = 2 Fairly Often = 3 Very Often = 4

1. In the **4 weeks**, how often have you been upset because of something that happened unexpectedly?
2. In the **4 weeks**, how often have you felt that you were unable to control the important things in your life?
3. In the **4 weeks**, how often have you felt nervous and “stressed”?
4. In the **4 weeks**, how often have you felt confident about your ability to handle your personal problems?
5. In the **4 weeks**, how often have you felt that things were going your way?
6. In the **4 weeks**, how often have you found that you could not cope with all the things that you had to do?
7. In the **4 weeks**, how often have you been able to control irritations in your life?
8. In the **4 weeks**, how often have you felt that you were on top of things?
9. In the **4 weeks**, how often have you been angered because of things that were outside of your control?
10. In the **4 weeks**, how often have you felt difficulties were piling up so high that you could not overcome them?
11. **Patient Health Questionnaire 9 (PHQ-9)**

The PHQ-9 is a widely used self-administered tool for screening, diagnosing, and monitoring the severity of depression [6]. It comprises nine items based on the Diagnostic and Statistical Manual of Mental Disorders 4th edition (DSM-IV) criteria. Total scores range from 0 to 27, reflecting symptom frequency over the previous two weeks, with higher scores denoting more severe depressive symptoms. Our research refers to the interpretation of PHQ-9 based on a previous study [7].

**The PHQ-9 questions**

Over the last 2 weeks, how often have you been bothered by any of the following problems?

- 1. Little interest or pleasure in doing things?
  2. Feeling down, depressed, or hopeless?
  3. Trouble falling or staying asleep or sleeping too much?
  4. Feeling tired or having little energy?
  5. Poor appetite or overeating?
  6. Feeling bad about yourself - or that you are a failure or have let yourself or your family down?
  7. Trouble concentrating on things, such as reading the newspaper or watching television?
  8. Moving or speaking so slowly that other people could have noticed? Or the opposite - being so fidgety or restless that you have been moving around a lot more than usual?
  9. Thoughts that you would be better off dead, or of hurting yourself in some way?

“Not at all= 0”, “Several days= 1”, “more than half the days= 2”, “nearly every day= 3”

1. **Generalized Anxiety Disorder (GAD-7)**

The GAD-7 is a self-reported scale used to assess the severity of generalized anxiety disorder symptoms [6]. It consists of seven items (S3) of the total scores ranging from 0 to 21, with higher scores indicating greater anxiety and severity measuring over the past two weeks. Our research refers to the GAD-7 interpretation based on a previous study [8,9].

**GAD-7 questions**

Over the last 2 weeks, how often have you been bothered by any of the following problems?

1. Feeling nervous, anxious, or on edge
2. Not being able to stop or control worrying
3. Worrying too much about different things
4. Trouble relaxing
5. Being so restless that it is hard to sit still
6. Becoming easily annoyed or irritable
7. Feeling afraid as if something awful might happen

Not at all = 0 Several days = 1 More than half the days = 2 Nearly every day = 3

**Section 3 COVID-19-related variables:** the measurement was adapted from the Vaccine Hesitancy Working Group as the measurement tool for assessment [10,11].

1. Level of fear of COVID-19 (Scoring 1= reported lowest fear , 10 = reported highest fear) categorized as
   - Low (1–4)
   - Moderate (5–7)
   - High (8–10)
2. Belief in the protective effect of the COVID-19 vaccine categorized as
   - Yes
   - No
   - Unsure (categorized as No)
3. The vaccine hesitancy: Have you ever been reluctant to get a COVID-19 vaccination? [12]
   - Yes [reported hesitancy]
   - No [no hesitancy]
   - Unsure (categorized as Yes, [reported hesitancy])

We collected data on COVID-19 vaccine hesitancy with response options: Participants who responded “Unsure” were categorized as having reported hesitancy. Therefore, both “Yes” and “Unsure” are defined as reported hesitancy, while “No” is defined as no hesitancy. [12]

**References**

[1] Cohen S. Perceived stress in a probability sample of the United States. 1988.

[2] Perceived Stress Scale n.d. https://uwm.edu/mcwp/wp-content/uploads/sites/337/2016/01/Perceived-Stress-Scale-Tip-Sheet.pdf (accessed January 25, 2025).

[3] Cohen S, Kamarck T, Mermelstein R. A global measure of perceived stress. J Health Soc Behav 1983:385–96.

[4] Sumi K. Reliability and validity of the Japanese version of the Perceived Stress Scale. Japanese Journal of Health Psychology 2006.

[5] Wongpakaran N, Wongpakaran T. The Thai version of the PSS-10: an investigation of its psychometric properties. Biopsychosoc Med 2010;4:1–6.

[6] Kroenke K, Spitzer RL, Williams JBW. The PHQ‐9: validity of a brief depression severity measure. J Gen Intern Med 2001;16:606–13.

[7] Kroenke K, Spitzer RL. The PHQ-9: A new depression diagnostic and severity measure. Psychiatr Ann 2002;32:509–15. https://doi.org/10.3928/0048-5713-20020901-06.

[8] Spitzer RL, Kroenke K, Williams JBW, Löwe B. A brief measure for assessing generalized anxiety disorder: the GAD-7. Arch Intern Med 2006;166:1092–7.

[9] Kroenke K, Spitzer RL, Williams JBW, Löwe B. The patient health questionnaire somatic, anxiety, and depressive symptom scales: a systematic review. Gen Hosp Psychiatry 2010;32:345–59.

[10] Domek GJ, O’Leary ST, Bull S, Bronsert M, Contreras-Roldan IL, Ventura GAB, et al. Measuring vaccine hesitancy: Field testing the WHO SAGE Working Group on Vaccine Hesitancy survey tool in Guatemala. Vaccine 2018;36:5273–81.

[11] World health organization. Report of the SAGE Working Group on Vaccine Hesitancy, Sage Report 2014. https://www.who.int/immunization/sage/meetings/2014/october/1_Report_WORKING_GROUP_vaccine_hesitancy_final.pdf (accessed June 7, 2021).

[12] Dubé E, Laberge C, Guay M, Bramadat P, Roy R, Bettinger J. Vaccine hesitancy: An overview. Hum Vaccin Immunother 2013;9:1763. https://doi.org/10.4161/HV.24657.
